# PPIA-coExp: Discovering Context-Specific Biomarkers Based on Protein–Protein Interactions, Co-Expression Networks, and Expression Data

**DOI:** 10.3390/ijms252312608

**Published:** 2024-11-24

**Authors:** Dongsheng Yan, Zhiyu Fan, Qianzhong Li, Yingli Chen

**Affiliations:** 1 School of Physical Science and Technology, Inner Mongolia University, Hohhot 010021, China; 31736009@mail.imu.edu.cn (D.Y.); 22146006@mail.imu.edu.cn (Z.F.); stchenyl@imu.edu.cn (Y.C.); 2 The State Key Laboratory of Reproductive Regulation and Breeding of Grassland Livestock, Inner Mongolia University, Hohhot 010021, China

**Keywords:** optimal model, biomarker, multi-omics data, human aging, Alzheimer’s disease

## Abstract

Identifying a small set of effective biomarkers from multi-omics data is important for the discrimination of different cell types and helpful for the early detection diagnosis of complex diseases. However, it is challenging to identify optimal biomarkers from the high throughput molecular data. Here, we present a method called protein–protein interaction affinity and co-expression network (PPIA-coExp), a linear programming model designed to discover context-specific biomarkers based on co-expressed networks and protein–protein interaction affinity (PPIA), which was used to estimate the concentrations of protein complexes based on the law of mass action. The performance of PPIA-coExp excelled over the traditional node-based approaches in both the small and large samples. We applied PPIA-coExp to human aging and Alzheimer’s disease (AD) and discovered some important biomarkers. In addition, we performed the integrative analysis of transcriptome and epigenomic data, revealing the correlation between the changes in gene expression and different histone modification distributions in human aging and AD.

## 1. Introduction

Biomarkers indicate molecular changes associated with illness. They help to predict disease development and identify specific molecular targets related to the disease [1,2]. In cancer, the use of optimal biomarkers for early detection and treatment is crucial for reducing mortality rates [3]. However, identifying effective biomarkers for complex diseases is challenging due to the high dimensionality of measured data [4,5]. Most conventional biomarker discovery methods mainly focus on quantifying individual protein expression and often overlook the interactions among them. For example, Yu et al. developed a computational approach based on expression enrichment and statistical significance for each gene in each tissue [6]. A novel feature selection method, ellipsoidFN [7], was developed for discovering node biomarkers based on linear programming. However, recent research suggests that genetic interactions among proteins play an important role in biological processes, and they found that different genes or proteins interact with each other to form network edges or pathways to carry out complex biological functions. Therefore, Ma X et al. proposed a computational method for discovering network biomarkers by combining network edge information likely protein–protein interaction data [8]. Zhang et al. introduced a vector representation in edge space based on the decomposed Pearson correlation coefficient (PCC) to identify gene pairs as edge biomarkers, which highlights the capacity and potential of edge information [9]. These results demonstrate the potential of utilizing information from edge features. One way to study edge information is to construct gene co-expression networks. Gene co-expression networks can be used for various applications that include functional gene annotation, identifying gene modules, and hub genes [10]. Gene co-expression networks are functionally context-specific networks, it is effectively able to identify correlations among proteins that are active in specific biological processes [11,12]. AD is a common chronic neurodegenerative disease. The hallmark pathology of AD involves the accumulation of amyloid-β plaques and tau tangles [13], which lead to neuronal cell death and the loss of cognitive function. Age-related neurodegeneration is the major factor in the pathogenesis of neurological disorders [14]. In addition, early-onset Alzheimer’s disease, which starts under the age of 65, is commonly referred to as “younger Alzheimer’s disease” [15,16]. Recent studies showed that there is no difference in time for diagnosis, functional dependence, and total neuropsychiatric symptoms (NPSs) between late-onset and early-onset AD [17]. This implies that the AD condition is similar and affects people in a similar way irrespective of age of onset. Although the association between aging and AD has been proposed, the changes in transcriptome and epigenomics underlying the similarities and differences are unclear. Epigenetic alterations can be caused by genetic and non-genetic factors such as environmental stimuli [18,19,20,21,22], but they can also represent potential therapeutically trackable players in aging and Alzheimer’s disease [23,24]. A recent study characterized the redistribution of histone 4 lysine 16 acetylation (H4K16ac) in the temporal cortex of 12 subjects with AD compared with the controls [25]. The study found genome-wide changes in the enrichment of H4K16ac with aging, as well as a loss of age-related protective epigenetic pathways in AD. Therefore, studying the epigenome may also be helpful for understanding the variations in histone modifications between aging and Alzheimer’s disease. In this paper, we propose an optimal method to identify a small set of biomarkers that maximize the distinctiveness between disease and normal samples. Unlike gene-node methods, PPIA-coExp can reveal important protein combinations and key genes. We constructed context-specific maps of active enhancers and different histone modifications in both aging and Alzheimer’s disease. These biomarkers and maps offer new insights into the relationship between aging and Alzheimer’s disease.

## 2. Results

### 2.1. Performance Evaluation of PPIA-coExp in ENCODE and TCGA-BRCA Database

**Figure 1:**
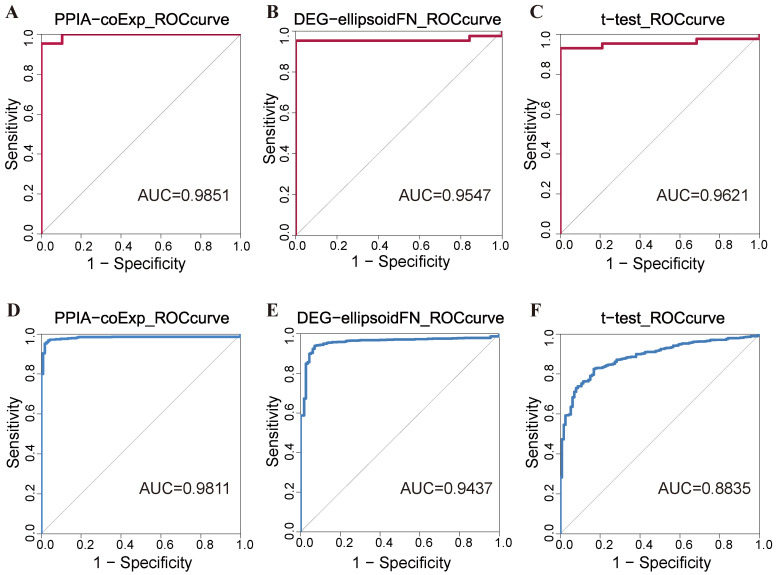
Performance evaluation of PPIA-coExp. ROC curves for the selected biomarker panel, each feature selection method exhibits predictive power, and PPIA-coExp outperforms node-based approaches in both ENCODE and TCGA-BRCA databases. ENCODE database: (**A**) the ROC curve of the PPIA-coExp model (AUC = 0.9851); (**B**) the ROC curve of the DEG-ellipsoidFN model (AUC = 0.9547); (**C**) the ROC curve of the *t*-test model (AUC = 0.9621). TCGA-BRCA database: (**D**) the ROC curve of the PPIA-coExp model (AUC = 0.9811); (**E**) the ROC curve of the DEG-ellipsoidFN model (AUC = 0.9437); (**F**) the ROC curve of the *t*-test model (AUC = 0.8835).

The PPIA-coExp model seeks to uncover the optimal biologically meaningful biomarkers by introducing network structure information between proteins in various biological processes. Figure 1A–C show the performance of PPIA-coExp, the traditional gene-node-based method, and the *t*-test statistical method in the public gene expression dataset for human breast tumors. The datasets were downloaded from the National Center for Biotechnology Information (NCBI) Gene Expression Omnibus (GEO) database with accession number GSE7904. There are 54,675 probes and 62 samples, including 19 normal samples and 43 disease samples. Genes with missing values and low information content (calculated by the entropy of the gene expression distribution [26]) were filtered out from the raw data. After these processes, we obtained a set of genes, including 712 genes. Then we mapped these genes to the human PPI network and co-expression network and obtained 4478 gene pairs. In the PPIA-coExp model, we identified 5 protein–protein interactions and 12 single genes, totaling 21 genes. In contrast, the traditional gene-node model identified 15 single genes. Using the *t*-test methods, we detected 34 significantly differentially expressed genes, based on their *p*-values being less than $5\times 10^{-5}$. We used a generalized linear model to predict the classification accuracy of disease samples versus normal samples using the biomarkers identified by each of these three methods. The results are shown in Table 1. Better results were obtained by using the PPIA-coExp model.

To further validate the generalizability of PPIA-coExp, gene expression data of breast cancer patients were downloaded from The Cancer Genome Atlas database (TCGA; https://portal.gdc.cancer.gov, accessed on 2 April 2024). The gene expression dataset consisted of 113 normal and 1118 tumor samples, genes with missing values, and low information content were filtered from the raw data. After the above processes, we finally obtained 861 genes. We applied PPIA-coExp, a gene-node-based method, and a *t*-test method to the TCGA-BRCA dataset. Better classification accuracies of breast cancer patients and normal people were obtained by using the PPIA-coExp method (Table 2). Additionally, we conducted a comparison with the MILP_k biomarker discovery method [27]. MILP_k identified 26 biomarkers in the TCGA-BRCA dataset. In the GSE7904 dataset, MILP_k identified 10 biomarkers. Then, we used a generalized linear model to predict the classification accuracy of disease samples and normal samples based on the biomarkers identified by MILP_k. MILP_k achieved an AUROC of 0.9329 in the TCGA-BRCA dataset and achieved an AUROC of 0.8986 in the GSE7904 dataset (Figure S1A,B). Further, we compared its performance with the random forest method, random forest achieved an AUROC of 0.9296 in the TCGA-BRCA dataset and achieved an AUROC of 0.9058 in the GSE7904 dataset (Figure S1C,D). These results demonstrated the generalizability of PPIA-coExp and indicated that the incorporation of context-specific network structures into the original gene expression data can improve performance (Figure 1D–F).

### 2.2. Transcriptomic Profiling Reveals the Differentially Expressed Genes Between Human Aging and Alzheimer’s Disease

**Figure 2:**
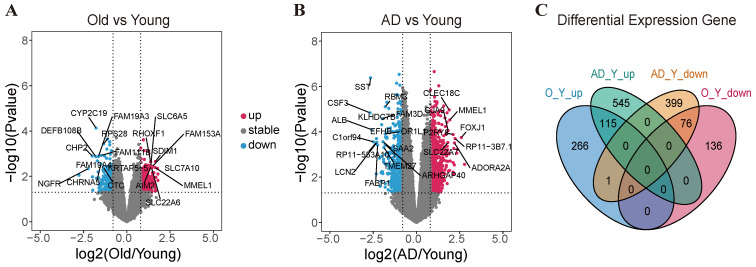

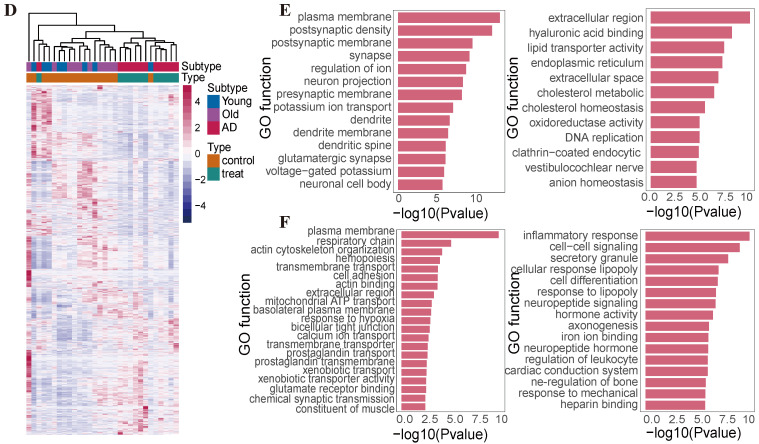
Transcriptomic analysis identifies DEGs between the younger-Old and younger-AD processes. Differentially expressed genes (DEGs) between (**A**) the younger-Old processes and (**B**) the younger-AD processes. Red, upregulated relative to younger groups; blue, downregulated. (**C**) Venn diagram of overlap of differentially expressed genes from the younger-Old processes and the younger-AD processes. (**D**) Hierarchical clustering analysis of differentially expressed genes involved in the younger-Old processes and the younger-AD processes. (**E**) GO enrichment analysis of differentially expressed genes in the younger-Old processes. (**F**) GO enrichment analysis of differentially expressed genes in the younger-AD processes.

To systematically investigate the changes in gene expression in aging and Alzheimer’s, we analyzed the RNA-seq data from a high-quality dataset (GSE153875). We conducted the differentially expressed gene analysis for the filtered genes (17,234) in the Old and AD groups compared with the younger groups. Figure 2A shows that a total of 382 genes were identified as upregulated in the Old groups compared with the younger groups, while 213 genes had significantly downregulated (*p* < 0.05). When gene expression profiles of AD were compared with the younger groups, a higher number of AD-specific differentially expressed genes were obtained in the AD groups compared with the younger groups. As shown in Figure 2B, 660 genes showed significantly upregulated in the AD groups compared with younger groups, while 476 genes showed significantly downregulated. This result suggests that the generation of AD is a more drastic process compared with aging. Hierarchical clustering of DEGs in aging and Alzheimer’s showed that the ‘Old’ and younger samples were clustered together, while AD samples were clearly separated from the younger samples (Figure 2D). Among aging and Alzheimer’s, we found that 191 DEGs were shared, with 115 genes commonly upregulated, and 76 genes commonly downregulated (Figure 2C). We then performed gene set enrichment analysis (GSEA) of the differentially expressed genes in aging and Alzheimer’s. In Alzheimer’s, we detected that the Alzheimer’s specific DEGs were mainly associated with Neuroactive ligand-receptor interaction and Alzheimer’s disease (Figure S2A,B). The aging-specific DEGs were enriched in gene sets involved in the synaptic vesicle cycle and Herpes simplex virus 1 infection (Figure S2C,D). GO ontology analysis showed that these genes in aging are associated with the plasma membrane, synapse, and DNA replication (Figure 2E). In Alzheimer’s disease, the genes are associated with signal transmission programs, including the plasma membrane, cell–cell signaling, and neuropeptide signaling. Some genes are also linked to the term “response to hypoxia”, which may induce the production of amyloid Aβ plaque (Figure 2F).

### 2.3. Genome-Wide Optimization Model for TF-Biomarkers and DEG-Biomarkers Identification

**Figure 3:**
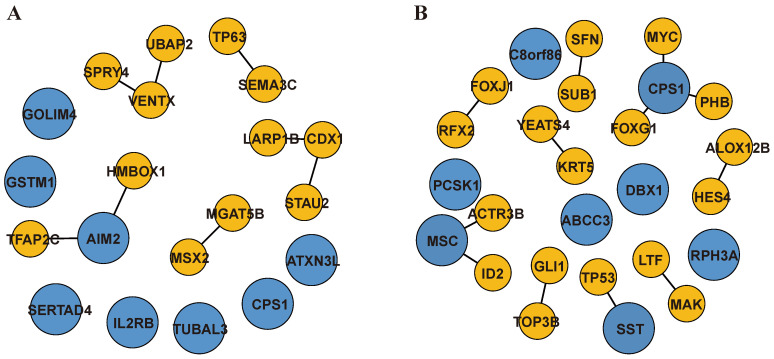
Biomarkers identified by PPIA-coExp. Biomarkers identified by PPIA-coExp in aging and Alzheimer’s. The blue and larger circles denote single-gene signatures identified by PPIA-coExp. The yellow and smaller circles denote genes involved in protein–protein interactions identified by PPIA-coExp. The presence of edges connecting genes represented by blue circles to those represented by yellow circles indicates that the blue-circle genes serve as both single-gene biomarkers and PPI biomarkers. (**A**) Includes 8 PPIAs and 8 single proteins in aging. (**B**) Includes 12 PPIAs and 8 single proteins in Alzheimer’s.

Sequence-specific core TFs are the key regulatory factors of eukaryotic gene regulation, the regulation of gene expression requires the specific binding of TFs at regulatory elements. To uncover the sequence-specific core TFs in aging and Alzheimer’s, we established a comprehensive database including DEGs, PPI network from the BioGRID database, and TF database. For each protein–protein pair, we extracted the differentially expressed TFs as one node of the PPIs, and the other node consisted of DEGs or genes associated with DEGs. Then, we obtained 39 differentially expressed TFs and 1866 DEGs and genes associated with DEGs, totaling 1905 genes in aging. In Alzheimer’s, 73 differentially expressed TFs and 2927 genes were selected. Finally, we identified 2765 PPIs in aging and 6567 PPIs in Alzheimer’s, respectively. These selected genes were extracted as input features for PPIA-coExp, and we applied the PPIA-coExp to human aging and Alzheimer’s. In aging, we identified 16 biomarkers, including 8 protein–protein interactions (consisting of 13 genes) and 8 single genes, totaling 20 genes (Figure 3A). There were six TFs (CDX1, VENTX, TP63, MSX2, HMBOX1, TFAP2C) involved in the eight protein–protein interactions, and the eight single signature genes were AIM2, GOLIM4, GSTM1, CPS1, SERTAD4, IL2RB, TUBAL3, and ATXN3L. Recent studies showed that the dynamic changes of HMBOX1 were closely associated with telomere shorting, making it a target for investing in the underlying mechanisms of aging and the etiology of aging-associated diseases [28]. The AIM2 gene was identified not only as a single-gene biomarker but also was found to be involved in PPI associated with HMBOX1. AIM2 was strongly associated with inflammation, which was proposed as an endogenous factor in aging, where chronic inflammation accelerates the senescence of immune cells, leading to weakened immune function and the inability to remove senescent cells [29].

In Alzheimer’s, we identified a set of biomarkers, including 12 protein–protein interactions (consisting of 21 genes) and 8 single signature genes (Figure 3B). The key TFs within 12 protein–protein interactions consisted of MSC, TP53, FOXJ1, MYC, YEATS4, FOXG1, PHB, HES4, TCF4, ID2, RFX2, SUB1, SETB2, LTF, and GLI1. The seven candidate signature genes were SST, RPH3A, PCSK1, CPS1, ABCC3, C8orf86, and DBX1. FOXG1 is a transcription factor mainly expressed in the brain and plays a critical role in the development and regionalization of the forebrain. Some research has shown that FOXG1 may be a critical node in the pathologic progression of AD and has the potential to serve as a therapeutic target [30]. SST is a neuropeptide hormone that contributes to the maintenance of blood–brain barrier (BBB) permeability and integrity by inhibiting Aβ-induced JNK phosphorylation and MMP2 expression [31].

To further investigate the heterogeneity of Alzheimer’s disease, we conducted a subgroup analysis of age. For all Alzheimer’s disease patients, we divided them into two groups. Group A consisted of AD patients over 70 years old, while Group B consisted of AD patients under 70 years old. We used DEG expression levels between Group A and Group B, the PPIA, and co-expression correlation coefficients as input features of PPIA-coExp. We identified 32 biomarkers, including 20 PPI biomarkers and 5 single-gene biomarkers. Then, we used the expression data of the 32 biomarkers to predict Group A and Group B through GLM in a 5-fold cross-validation. We found that PPIA-coExp achieved a commendable AUROC of approximately 0.9218. These results demonstrate that PPIA-coExp can effectively identify biomarkers between age subgroups in AD. The results for the age subgroup analysis in AD are depicted in Figure S3.

### 2.4. Difference in Histone Modification Distribution in the Younger, Old, and AD Groups

To reveal the differences in histone modifications between aging and Alzheimer’s, we calculated the average histone modification signals (H3K27ac, H3K9ac, H3K4me1, H3K122ac) in 100 bins, each spanning 500 kb upstream and downstream flanking TSS across all 17,234 protein-coding genes and the differentially expressed genes. The signals of H3K9ac and H3K27ac within each bin were clearly increased in the AD groups, while the signals were weakest in the Old groups and the signal of H3K9ac was globally increased compared to H3K27ac in three contexts for the 17,234 protein-coding genes (Figure S4A,B). In contrast, the signal of H3K122ac was the lowest in the AD groups and the most robust in the Old groups (Figure S4C).

**Figure 4:**
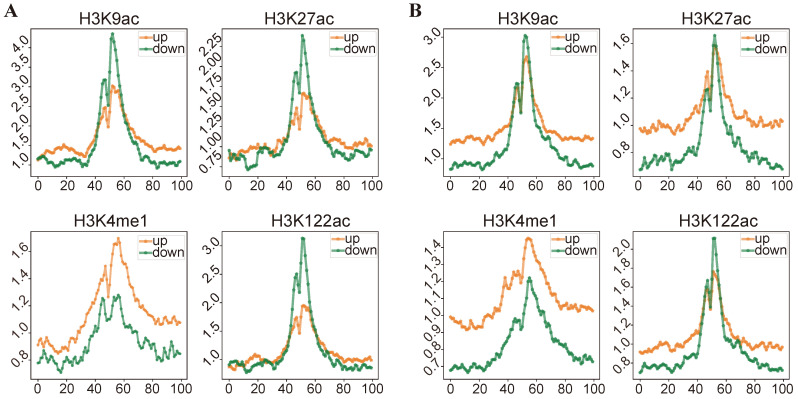

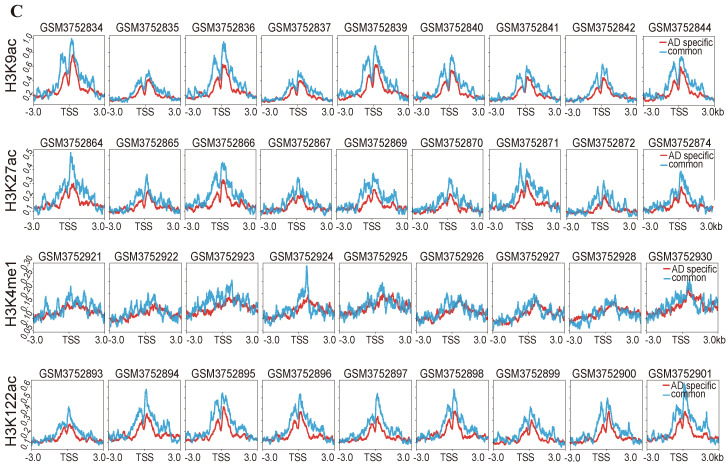
The signal distribution of 4 HMs in DEGs in aging and Alzheimer’s. (**A**) In aging, four histone modification distributions of upregulated genes and downregulated genes in the Old groups. (**B**) In Alzheimer’s, four histone modification distributions of upregulated genes and downregulated genes in the AD groups. (**C**) Four histone modification signals relative to ±3 kb of the transcription factor (TSS) between AD-specific downregulated genes and common downregulated genes in the AD groups.

The histone modification signal distributions of upregulated and downregulated genes in Alzheimer’s and aging are shown in Figure 4A,B. In the AD groups, we observed that the four histone modification signals from the 50th to the 10th bins upstream of the TSS were significantly increased in upregulated genes compared to downregulated genes. In the Old groups, the four histone modification signals in the same regions (from the TSS upstream 50th bins to TSS upstream 10th bins) remained relatively stable. However, significant changes in H3K9ac, H3K27ac, and H3K122ac were noted from the TSS upstream 5th bins to the TSS downstream 5th bins. These findings suggest that histone modifications tend to regulate distal regions in Alzheimer’s, whereas, in aging, they predominantly affect regions proximal to the TSS.

To further explore the similarities and differences between Alzheimer’s and aging, we divided the differentially expressed genes into six categories, including AD-specific upregulated genes, Old-specific upregulated genes, common upregulated genes, AD-specific downregulated genes, Old-specific downregulated genes, and common downregulated genes. We used deepTools [32] to identify the difference in histone modification signals between the six categories of genes. Figure 4C shows that there were apparent decreases in AD-specific downregulated genes compared to common downregulated genes in the four histone modifications. However, for the comparison of the other specific DEGs and common DEGs, there were no significant changes in their corresponding histone modification levels (Figures S5–S7). These findings demonstrate a significant association between the histone modification of AD-specific downregulated genes and their regulatory mechanisms. Additionally, we further investigated the relationship between abnormal histone modifications and the dysregulation of biomarker genes. Specifically, for the upregulated biomarker genes, we observed significantly higher histone modification signals compared to all upregulated genes in H3K9ac, H3K27ac, and H3K122ac (Figure S8). Similarly, downregulated biomarker genes exhibited significantly lower histone modification signals compared to all downregulated genes flanking the TSS loci in the H3K9ac, H3K27ac, and H3K122ac (Figure S9).

### 2.5. AD-Specific and Old-Specific Active Enhancers

**Figure 5:**
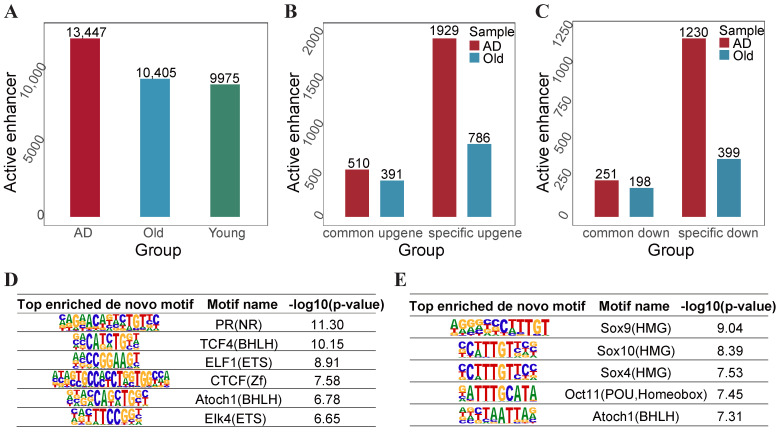
Identification of context-specific active enhancers. (**A**) The number of active enhancers in the younger, Old, and AD groups. (**B**) The total number of active enhancers between specific specific upregulated genes and common upregulated genes in aging and Alzheimer’s. (**C**) The total number of active enhancers between specific specific downregulated genes and common downregulated genes in aging and Alzheimer’s. (**D**) The transcription factors and binding motif identified at the H3K37ac-enriched active enhancers in the AD groups relative to younger groups. (**E**) H3K27ac-enriched active enhancers in the Old groups relative to younger groups.

Aberrant enhancer activity also plays a key role in the changes in gene expression that contribute to both aging and Alzheimer’s. We identified high-confidence H3K27ac peaks by intersecting the peaks from replicates in the younger, Old, and AD groups. The intersections between high-confidence peaks and putative enhancer regions from the Roadmap Epigenomics Project were defined as ‘high-confidence’ active enhancers. As shown in Figure 5A, we obtained 13,447, 10,405, and 9975 active enhancers in the AD groups, Old groups, and younger groups. Our results show that AD-specific upregulated genes have the largest number of active enhancers and the majority of AD-specific upregulated genes are associated with 2 to 8 enhancers, with an average of 5.73 enhancers per gene (Figure 5B,C).

Furthermore, in order to identify the transcription factors that potentially regulate gene expression in Alzheimer’s and aging, we performed motif enrichment analysis in the H3K27ac enriched enhancer regions for the AD groups and the Old groups compared to the younger groups. In Alzheimer’s, the NR, BHLH, ETS, Zf, and HTH families were the top motifs enriched with H3K27ac peaks (Figure 5D). Similarly, the HMG, POU, homeobox, and BHLH families were enriched in aging (Figure 5E). Then, we executed motif enrichment analysis in the active enhancer regions for the AD groups relative to the Old groups, identifying the ETS, homeobox, EBF, HLH, and Zf families. Notably, we found the neuron-associated TF, phox2a, inside the homeobox family for the AD groups.

## 3. Discussion

In summary, we developed PPIA-coExp, an optimal model to integrate the multiple-level dataset. Most existing biomarker discovery models primarily focus on single-gene biomarkers. Our major contribution is the introduction of functionally context-specific network structure information based on PPIA and co-expression networks. The PPIA-coExp model utilizes the law of mass action (by calculating the product of gene expression levels corresponding to the two proteins in a PPI) to embed gene expression data into the PPI network, thereby imparting a context-specific characteristic to the network. This approach overcomes the static limitations of traditional PPI networks and allows them to dynamically reflect specific biological contexts. PPIA-coExp revealed context-specific functional associations among genes by integrating the gene co-expression network into the PPI network and enabling more precise identification of PPI biomarkers relevant to specific biological contexts. Through validation using the ENCODE and TCGA-BRCA datasets, PPIA-coExp demonstrated superior performance. Furthermore, we applied PPIA-coExp to human aging and Alzheimer’s, identifying some key TFs. Specifically, ABO has demonstrated anti-aging effects in mouse models by regulating key cellular mechanisms such as lysosomal acidification and microfilament organization. ABO treatment elevated HMBOX1 protein levels, which supported microfilament homeostasis and reduced senescence phenotypes. HMBOX1 likely functions downstream of ANXA7, interacting with cytoskeletal components to mediate these protective effects against aging [33]. Emerging evidence suggests that the AIM2 inflammasome plays a critical role in the pathogenesis of post-stroke cognitive impairment (PSCI) through inflammatory processes and pyroptosis. In the middle cerebral artery occlusion (MCAO)-induced PSCI mouse model, AIM2 expression was upregulated in microglia and endothelial cells, correlating with cognitive deficiency observed at 28 days post-stroke. The knockout of AIM2 in mice significantly improves cognitive function and reverses brain volume loss in the hippocampus [34]. FOXG1 plays a pivotal role in counteracting Alzheimer’s disease (AD) pathology by modulating the neuronal cell cycle. In the mice AD model, FOXG1 expression was significantly reduced alongside increased neuronal apoptosis and amyloid-β accumulation. FOXG1 appears to block these effects by inhibiting cell cycle reentry through pathways involving p21 and cyclin. Within an in vivo mice Aβ amyloidosis model, SST deficiency led to a modest increase in cortical Aβ plaque density, western blot analyses of whole brain extracts indicated that SST interferes with early steps of Aβ assembly that manifest in the appearance of sodium dodecyl sulfate (SDS)-stable smears of 55–150 kDa in SST null brain samples [35].

Histone signal distribution analysis of H3K9ac, H3K27ac, and H3K122ac revealed H3K9ac and H3K27ac preferential increases in AD, and H3K122ac exhibited an opposite trend. The dysregulation of epigenetic modifications accompanies changes observed in RNA-seq data, histone signals associated with DEGs expert influence in distinct regions between aging and Alzheimer’s. To explore the fine relationships, we divided the DEGs into six categories and found that AD-specific downregulated genes have a significant negative correlation with the four histone modifications. Our research revealed distinct histone modification patterns associated with upregulated biomarker genes and downregulated biomarker genes. Specifically, upregulated biomarker genes displayed higher histone modification signals in H3K9ac, H3K27ac, and H3K122ac compared to all upregulated genes, whereas downregulated biomarker genes exhibited consistently lower histone modification signals in H3K9ac, H3K27ac, and H3K122ac flanking the TSS loci. These findings underscore the distinct epigenetic landscape of biomarker genes and provide insights into the potential mechanisms underlying their transcriptional regulation.

Our work provides new insight into the relationship between histone modification distributions and gene expression in aging and Alzheimer’s. Moreover, we present a general optimal model to identify context-specific biomarkers. This study has primarily focused on transcription factors as key regulatory factors. However, other types of regulatory factors, such as RNA-binding proteins and histone-modifying enzymes, also play important roles in gene expression and cellular processes associated with aging and Alzheimer’s disease. In the future, we aim to expand our research to incorporate these additional regulatory factors, providing a more comprehensive understanding of the molecular mechanisms in aging and Alzheimer’s disease.

## 4. Materials and Methods

### 4.1. Estimating the Protein–Protein Interaction Affinity by the Law of Mass Action

PPI interactions and the related networks play a fundamental role in the vast majority of biological functions and processes, which can be regarded as biomarkers for diagnosing disease [36]. Here, protein–protein interactions were constructed by using the public database, Biological General Repository for Interaction Datasets (BioGRID). We estimate the protein–protein interaction affinity (PPIA) from gene expression data by employing the law of mass action [37,38]. The affinity between protein A and protein B can be given by the following:

$$\rho_{AB}=\alpha {\left[A\right]}^{a}{\left[B\right]}^{b}$$

where $\rho_{AB}$ is the affinity between protein A and protein B, the concentrations of protein A and protein B are [A] and [B], which are in a proportional relationship to their corresponding mRNA expression levels. α is the reaction constant, and the stoichiometric coefficients of protein A and protein B are represented by *a* and *b*, respectively. The empirical constants *a*, *b*, and α are determined by the chemical properties of protein A and protein B.

Within class *K*, the average affinity level $a_{i}^{k}=\frac{\sum_{j\in I^{k}}\left(x_{ju}\cdot x_{jv}\right)}{m^{k}}$ characterizes the *i*-th protein–protein interaction involving protein *u* and protein *v*. The set $I^{k}$ consists of $m^{k}$ samples that belong to class *K*, encompassing a total number of samples. $a_{ji}=x_{ju}\cdot x_{jv}$ denotes the affinity levels of the *i*-th protein–protein interaction between protein *u* and protein *v* in the *j*-th sample. $x_{ju}$ and $x_{jv}$ denote the expression levels of the *u*-th gene and the *v*-th gene in the *j*-th sample, respectively.

### 4.2. Construct Context-Specific Gene Co-Expression Networks

Gene co-expression networks can identify which genes tend to show coordinated expression patterns across a group of samples [39,40]. This can be represented by a gene–gene correlation matrix, where each node represents a gene and each edge represents the presence and strength of the gene–gene relationship. To identify specific biomarkers in the younger-Old processes and younger-AD processes, we introduce the gene co-expression networks to enhance PPIA specificity as follows:

$$B_{uv}=1-\frac{6\sum_{l=1}^{m}{\left[rg\left(x_{lu}\right)-rg\left(x_{lv}\right)\right]}^{2}}{m(m-1)}$$

$$B_{uv}=I_{i}\left(\delta =k\right)=\left\{\begin{array}{cc} 0 & \mathrm{if}\;B_{uv}<k\\ B_{uv} & \mathrm{if}\;B_{uv}\geq k \end{array}\right.$$

where $x_{lu}$ and $x_{lv}$ denote the expression levels of the *u*-th gene and the *v*-th gene in the *l*-th sample, respectively. The $rg(x_{lu})$ and $rg(x_{lv})$ are the rank numbers of $x_{lu}$ and $x_{lv}$. The term $I_{i}\left(\delta =k\right)=B_{uv}$ denotes the *i*-th pair’s co-expression coefficient involving the *u*-th gene and the *v*-th gene. When δ=k is the cutoff, if $B_{uv}$ is greater than *k*, it indicates that the protein–protein pair has a strong relationship in a specific context. Otherwise, we assume there is no binding relationship between protein *u* and protein *v*, and $B_{uv}$ is set to 0.

### 4.3. Overview of the PPIA-coExp

For any protein–protein interaction, we can approximate the protein–protein interaction affinity by the law of mass action, however, the direct calculation of affinity by PPIA limits context specificity. Several studies have demonstrated that the co-expression network is context-specific and used only differential co-expression networks to identify networks unique to specific tissues or disease states. Therefore, there is considerable interest in context-specific PPIA design strategies that consider both PPIA and context-specific co-expression network.

**Figure 6:**
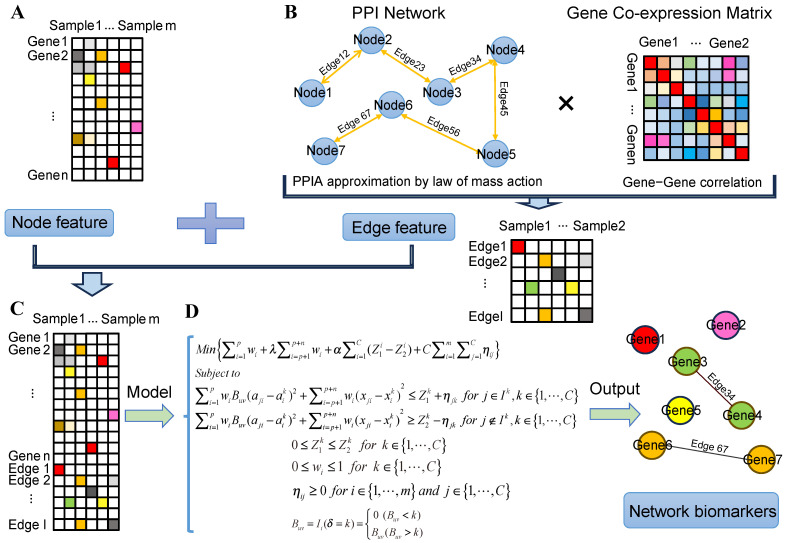
Overview of PPIA-coExp. The framework of PPIA-coExp is illustrated from (**A**–**D**). (**A**) Gene expression matrix where rows represent genes, and columns represent samples. (**B**) Protein–protein interaction (PPI) data and co-expression matrix. Gene expression values are embedded into the PPI network using the law of mass action, resulting in protein–protein interaction affinity (PPIA); each PPIA is further refined by incorporating the co-expression correlation coefficient, producing a context-specific PPIA expression matrix. (**C**) The PPIA expression matrix and the gene expression matrix are combined to create a unified feature matrix. This matrix serves as the final input to the PPIA-coExp model, capturing both node-level (gene-specific) and edge-level (interaction-specific) features. (**D**) We adopt a linear programming framework, embedding an inner and outer radius of the ellipsoid, by minimizing the distance of samples from its class center and maximizing the distance between ellipsoids. The model output includes sets of biomarkers, which comprise node features and edge features.

We propose an optimal model to identify biomarkers by integrating gene expression data, PPI network, and co-expression network (Figure 6). In the PPIA-coExp, each cluster of samples was theoretically represented by an ellipsoid, which can effectively capture nonlinear relationships among mixed samples in high-dimensional data. The objective of this optimal model focuses on finding a minimal possible context-specific biomarkers to maximize the distance between different clusters.

Suppose we have a gene expression matrix $X_{m\times n}$, in which the expression of *n* genes are measured for *m* samples and $x_{ij}$ denotes the expression levels of the *j*-th gene in the *i*-th sample. As a result, the protein–protein interaction affinity matrix can be derived as $A_{m\times p}$, in which the affinity of *p* protein–protein interactions was calculated for *m* samples. The gene co-expression network can be denoted as $B_{n\times n}$, where we integrate PPIA and co-expression network to compute context-specific PPIA by $B_{n\times n}\times A_{m\times p}$. Overall, the optimization model can be described as follows:

(1) $$\mathrm{Min}\left(f\right)=\min\left\{\sum_{i=1}^{p}w_{i}+\lambda \sum_{i=p+1}^{p+n}w_{i}+\alpha \sum_{i=1}^{C}\left(Z_{1}^{i}-Z_{2}^{i}\right)+C\sum_{i=1}^{m}\sum_{j=1}^{C}\eta_{ij}\right\}$$

subject to the following:

(2) $$\begin{array}{cc} \sum_{i=1}^{p}w_{i}B_{uv}{\left(a_{ji}-a_{i}^{k}\right)}^{2}+\sum_{i=p+1}^{p+n}w_{i}{\left(x_{ji}-x_{i}^{k}\right)}^{2} & \leq Z_{1}^{k}+\eta_{jk}\;\mathrm{for}\;j\in I^{k},k\in \left\{1,\ldots ,C\right\} \end{array}$$

(3) $$\begin{array}{cc} \sum_{i=1}^{p}w_{i}B_{uv}{\left(a_{ji}-a_{i}^{k}\right)}^{2}+\sum_{i=p+1}^{p+n}w_{i}{\left(x_{ji}-x_{i}^{k}\right)}^{2} & \geq Z_{2}^{k}-\eta_{jk}\;\mathrm{for}\;j\notin I^{k},k\in \left\{1,\ldots ,C\right\} \end{array}$$

(4) $$0\leq Z_{1}^{k}\leq Z_{2}^{k}\;\mathrm{for}\;k\in \left\{1,\ldots ,C\right\}$$

(5) $$0\leq w_{i}\leq 1\;\mathrm{for}\;i\in \left\{1,\ldots ,p+n\right\}$$

(6) $$\eta_{jk}\geq 0\;\mathrm{for}\;i\in \left\{1,\ldots ,m\right\},j\in \left\{1,\ldots ,C\right\}$$

(7) $$B_{uv}=I_{i}\left(\delta =k\right)=\left\{\begin{array}{cc} 0 & \mathrm{if}\;B_{uv}<k\\ B_{uv} & \mathrm{if}\;B_{uv}\geq k \end{array}\right.$$

In the objective function *f*, $\sum_{i=1}^{p}w_{i}+\lambda \sum_{i=p+1}^{p+n}w_{i}$ denotes the first term of the objective function *f* in the optimization model. It represents the total weight of the selected single genes and protein–protein interactions. By minimizing it, we aim to enhance the model interpretability by selecting the smallest set of protein–protein interactions and single genes as biomarkers. $Z_{1}^{k}$ and $Z_{2}^{k}$ are variables that define the inner and outer radius of the ellipsoid representing class *K*. We minimized the second-term $\sum_{i=1}^{C}\left(Z_{1}^{i}-Z_{2}^{i}\right)$ of *f* in order to enlarge the difference between the inner and outer radius of the ellipsoid. $\eta_{ij}$ is a slack variable to tolerate the data errors. The third term $\sum_{i=1}^{m}\sum_{j=1}^{C}\eta_{ij}$ denotes the total classification error for all the samples and should be minimized to achieve the highest classification accuracy.

In the constraint formulas, $a_{i}^{k}$ denotes the average affinity of the *i*-th PPI within class *K*, while $a_{ji}$ represents the affinity of the *i*-th PPI in the *j*-th sample. The first term of Equation (2), $\sum_{i=1}^{p}w_{i}B_{uv}{\left(a_{ji}-a_{i}^{k}\right)}^{2}$, represents high specificity, and can be used to identify potential context-specific protein–protein interactions.

$x_{i}^{k}=\frac{\sum_{j\in k}x_{ji}}{m^{k}}$ represents the average expression levels of the *i*-th gene in class *K* for all samples, where $x_{ji}$ denotes the expression value of the *i*-th gene in the *j*-th sample. In the second term of Equation (2), $\sum_{i=p+1}^{p+n}w_{i}{\left(x_{ji}-x_{i}^{k}\right)}^{2}$, *n* denotes the number of selected genes, and $w_{i}$ for i=p+1,p+2,…,p+n are weights for each gene to be selected as node biomarkers.

λ, α, and *C* are three parameters introduced to balance the three terms and unify them into a single objective function. In practice, we hypothesize that protein–protein interactions and single genes have the same importance as biomarkers, so the parameter λ can be set to 1. The remaining parameters, α and *C*, were determined using grid search methods. We tested α = 0.02, 0.1, 0.5,and1, and C = 0.1, 1, 10, and 100. In this model, a sufficiently small α or a sufficiently larger *C* will generate a trivial zero solution, implying that no biomarkers are identified. A smaller α corresponds to fewer biomarkers, whereas a larger *C* leads to fewer classification errors. Accordingly, we selected the parameter pair (α=0.1, C=100), as it achieves a non-trivial solution while maintaining a relatively smaller α and a larger *C*. This combination represents the optimal parameter configuration in the PPIA-coExp.

### 4.4. Evaluation Metrics

We applied the 10-fold cross-validation method to evaluate the performance of the PPIA-coExp model. The datasets were randomly divided into 10 equally sized groups, with 9 groups used as training sets each time, and the remaining group used as the test set. Sensitivity $S_{n}$, specificity $S_{p}$, and accuracy ACC were calculated as follows:

$$S_{n}=\frac{TP}{TP+FN}$$

$$S_{p}=\frac{TN}{TN+FP}$$

$$ACC=\frac{TP+TN}{TP+TN+FP+FN}$$

where TP denotes the number of correctly identified disease samples, TN denotes the number of correctly identified normal samples, FP denotes the number of normal samples identified as disease samples, and FN denotes the number of disease samples identified as normal samples.

### 4.5. Data Collection and Pre-Processing

We collected the histone modification data (H3K9ac, H3K27ac, H3K122ac, H3K4me1) and RNA-seq data from the NCBI GEO database (GSE153875, https://www.ncbi.nlm.nih.gov/geo/, accessed on 20 December 2023), to investigate the changes in gene expression and histone modification signal distributions between the younger-Old processes and the younger-AD processes. We also downloaded public H3K27ac and H3K4me1 chip-seq data from Roadmap to annotate putative enhancer regions. The protein–protein interaction data comes from the BioGRID database (https://thebiogrid.org, accessed on 1 March 2024).

RNA-seq FASTQ format data were processed using fastp (v0.20.0) [41] with default parameters. Clean reads were aligned to the human reference genome (hg19) using HISAT2 (v2.2.1) [42] with the “–dta-cufflinks” parameters. Afterward, we used SAMtools (v1.9) [43] to filter out sequences with low alignment quality. Transcripts per million (TPM) values were calculated and normalized by using featureCounts (v2.0.6) [44] with default parameters. The replicates where the ratio of maximum to minimum TPM was larger than 100 were also filtered. Finally, 17,234 protein-coding genes remained for subsequent analysis. Histone Chip-seq reads were trimmed for Illumina adapter sequences and then mapped to the hg19 genome by using Bowtie 2 (v2.5.1) [45]. Peaks were called by MACS2 (v2.2.7.1) [46] with default parameters.

### 4.6. Differentially Expressed Genes and Pathway Enrichment Analysis

The limma package was used to identify differentially expressed genes during aging (younger versus Old) and Alzheimer’s (younger versus AD). Genes with $\log_{2}\left(\mathrm{FC}\right)>0.8$ and adjusted *p*-value < 0.05 were identified as upregulated genes, while genes with $\log_{2}\left(\mathrm{FC}\right)<-0.8$ and adjusted *p*-value < 0.05 were identified as downregulated genes. For the differentially expressed genes in aging and Alzheimer’s, we performed gene set enrichment analysis (GSEA) by using the R package ChIPseeker (v1.26.0) [47]. The top enriched pathways were selected.

### 4.7. The Histone Modification Levels Flanking the Transcription Start Site (TSS)

In order to investigate the signal distributions of four HMs flanking the TSS in all 17,234 genes and the differentially expressed genes (DEGs) for the younger, Old, and AD samples, we divided the upstream and downstream 5 kb of the TSS for all genes into 100 bins. Similarly, there were 60 bins corresponding to ±3 kb of the TSS in the DEGs. Then, the signals of HMs were normalized as follows:

$$H_{ijl}^{k}=\frac{h_{ijl}^{k}\times 10^{9}}{h_{kl}\times L_{j}}$$

$$H_{ij}^{k}=\frac{1}{m}\sum_{l=1}^{m}H_{ijl}^{k}$$

where $H_{ij}^{k}$ denotes the average signal level of the *k*-th HM in the *j*-th bin of the *i*-th gene. $h_{ijl}^{k}$ denotes the total read counts of the *k*-th HM located in the *j*-th bin of the *i*-th gene in the *l*-th samples, $h_{kl}$ denotes the sequencing depth of the *k*-th HM in the *l*-th samples. $L_{j}$ denotes the length of the *j*-th bins, and $10^{9}$ is used to balance the consistent magnitude of the TPM.

### 4.8. Enhancer Annotation and Motif Enrichment

For each gene, the putative enhancers were annotated by using the peaks of H3K4me1 and H3K27ac from Roadmap (E081) in the ±200 kb regions flanking the transcription start site (TSS). For the younger, Old, and AD populations, each condition has multiple samples. We merged the histone modification peaks of replicates using BEDTools (v2.26.0). The candidate active enhancers were calculated by using the putative enhancer regions and merged H3K27ac peaks through the intersect function in the younger, Old, and AD populations.

Motif enrichment analyses were performed for both Old-specific and AD-specific active enhancer regions using HOMER (v4.11.1) with parameters “-size 1000 -mask” setting. Transcription factors (TFs) with high expression levels in Old-specific and AD-specific enhancers, as well as significant H3K27ac enrichment, were identified as specific active motifs.

### 4.9. Hierarchical Clustering

We performed the hierarchical clustering for the expression data of the differentially expressed genes, respectively. Hierarchical clustering was implemented using the clustering distance (Pearson correlation) and the complete linkage. The heatmap was plotted with R package pheatmap (v1.0.12).

## 5. Conclusions

Our work provides new insight into the relationship between histone modification distributions and gene expression in aging and Alzheimer’s. Moreover, we present a general optimal model to identify context-specific biomarkers. In the future, we will integrate other omics data, likely Hi-C and multiple node specificity-designed methods to reveal heterogeneous network modules.

## Figures and Tables

**Table 1:** Performance comparison among various methods in the ENCODE dataset through the 10-fold cross-validation test.

Model	S_n_	S_p_	ACC	AUC
Gene-node	0.7895	0.9535	0.9032	0.9547
*t*-test	0.8947	0.9302	0.9194	0.9621
PPIA-coExp	0.9474	0.9535	0.9516	0.9851

**Table 2:** Performance comparison among various methods in the TCGA-BRCA datasets through the 10-fold cross-validation test.

Model	S_n_	S_p_	ACC	AUC
Gene-node	0.9027	0.9425	0.9326	0.9437
*t*-test	0.4124	0.9737	0.8765	0.8835
PPIA-coExp	0.8850	0.9762	0.9650	0.9811

## Data Availability

All the data for this study were obtained from publicly available databases such as TCGA (https://portal.gdc.cancer.gov/, accessed on accessed on 2 April 2024) and GEO (https://www.ncbi.nlm.nih.gov/geoprofiles/, accessed on 20 December 2023).

## Supplementary Materials

The following supporting information can be downloaded at https://www.mdpi.com/article/10.3390/ijms252312608/s1.

## Abbreviations

The following abbreviations are used in this manuscript:

PPIA-coExp	protein–protein interaction affinity–co-expression network
PPI	protein–protein interaction
PPIA	protein–protein interaction affinity
AD	Alzheimer’s disease
PCC	Pearson correlation coefficient
BioGRID	Biological General Repository for Interaction Datasets
Sensitivity	$S_{n}$
Specificity	$S_{p}$
Accuracy	ACC
RNA-Seq	RNA sequencing
TPM	transcripts per million
GSEA	gene set enrichment analysis
DEGs	differentially expressed genes
HMs	histone modifications
TSS	transcription start site
TFs	transcription factors
