# Supplementary material for: PPIA-coExp: Discovering Context-Specific Biomarkers Based on Protein–Protein Interactions, Co-Expression Networks, and Expression Data
Source: Int J Mol Sci. 2024 Nov 24;25(23):12608. doi: 10.3390/ijms252312608 (PMC11641600; doi:10.3390/ijms252312608)
Supplement: Supplementary file 1 [file ijms-25-12608-s001.zip › Supplement_Figure.pdf]

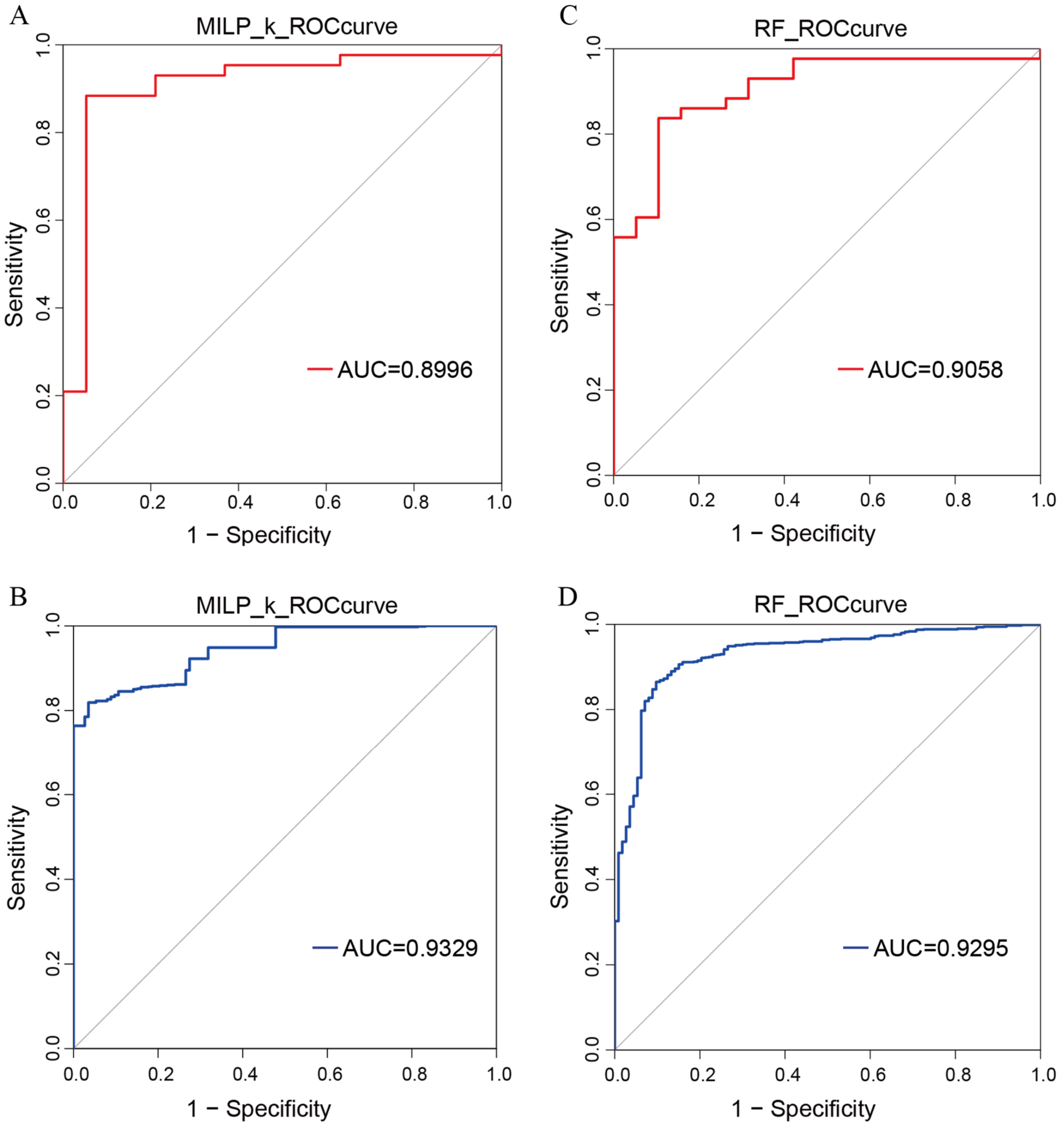

Figure S1. ROC curves for the selected biomarker panel of MILP_k and Random Forest in both GSE7904 and TCGA-BRCA database. MILP_k(A) The ROC curve in the GSE79047(B) The ROC curve in the TCGA-BRCA. Random Forest(C) The ROC curve in the GSE79047 (D) The ROC curve in the TCGA-BRCA.

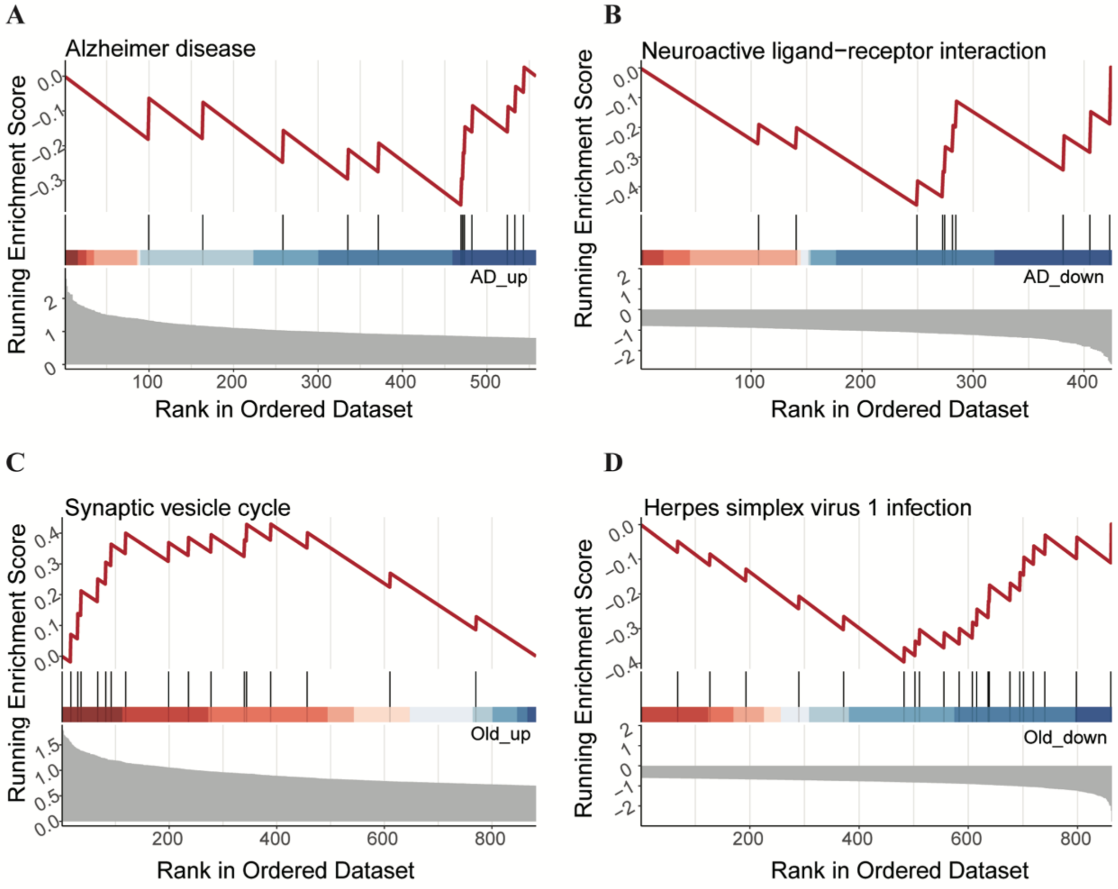

Figure S2. GSEA analysis for differentially expressed genes associated with younger-AD process and younger-Old process (A)up-regulated genes in AD groups (B)down-regulated genes in AD groups (C)up-regulated genes in Old groups (D)down-regulated genes in Old groups.

A

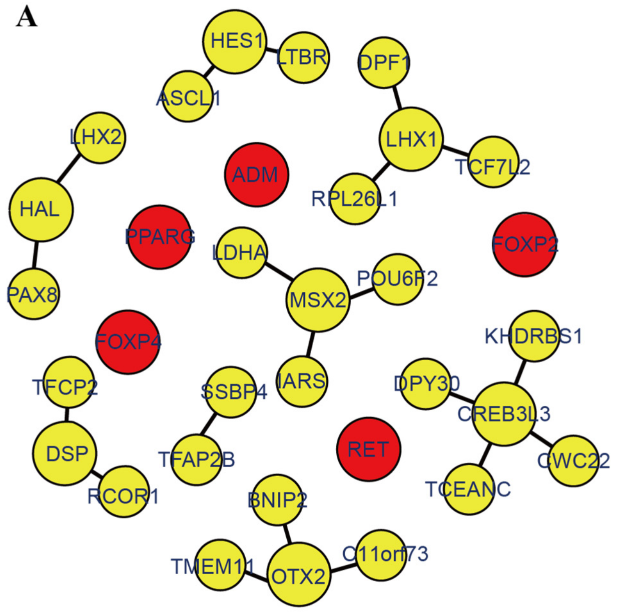

B

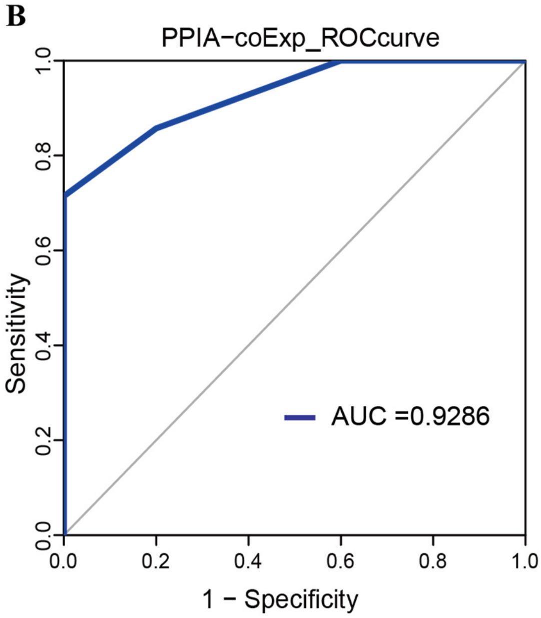

Figure S3. The analysis of age subgroups by PPIA-coExp. Alzheimer's disease (AD) patients were divided into two age subgroups based on the threshold of 70 years. (A) Age subgroup specific biomarkers were identified by PPIA-coExp, including 20 PPIs and 5 single genes. (B) ROC curves for the selected biomarker panel of the PPIA-coExp.

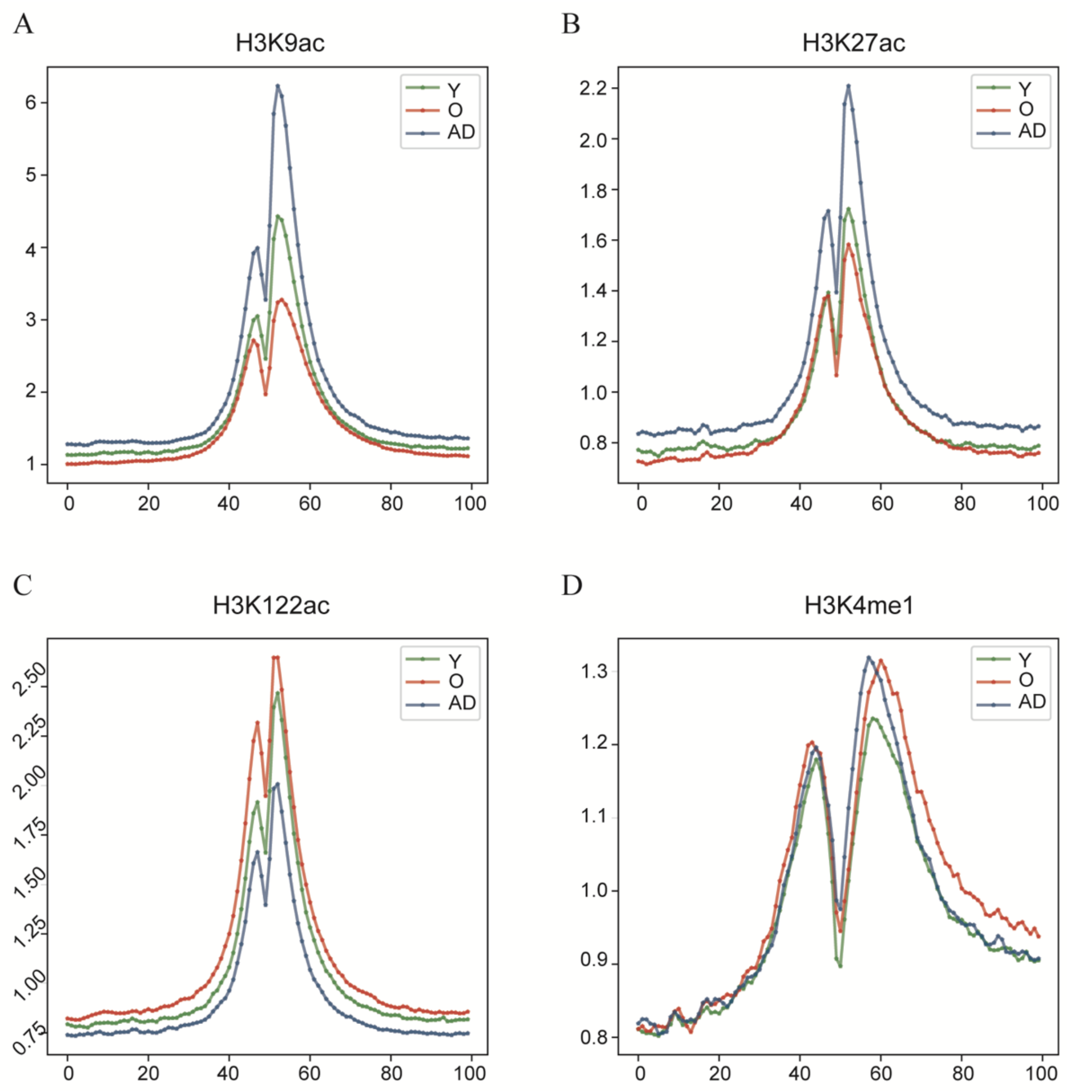

Figure S4. The average signal distribution of four HMs in all 17234 protein-coding genes in the 5kb regions of upstream and downstream flanking the TSS (A) H3K9ac (B) H3K27ac (C) H3K122ac (D) H3K4me1

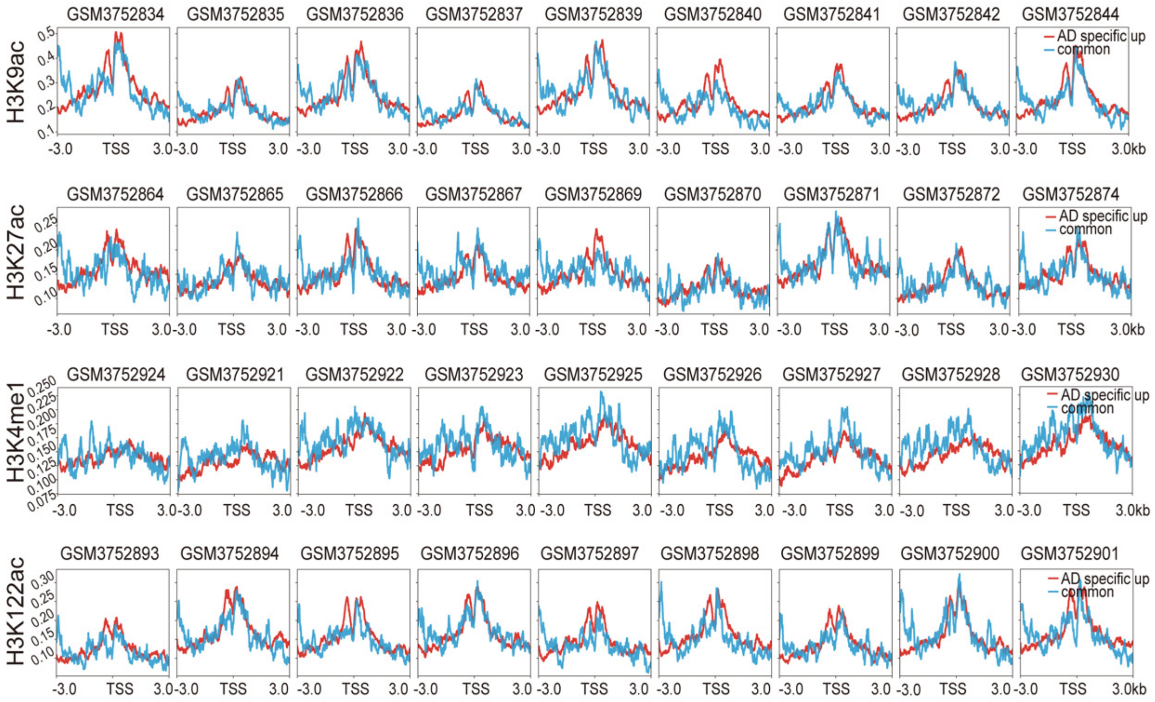

Figure S5. Four HMs signal distributions relative to ±3kb of the TSS between AD specific up-regulated genes and common up-regulated in the AD groups

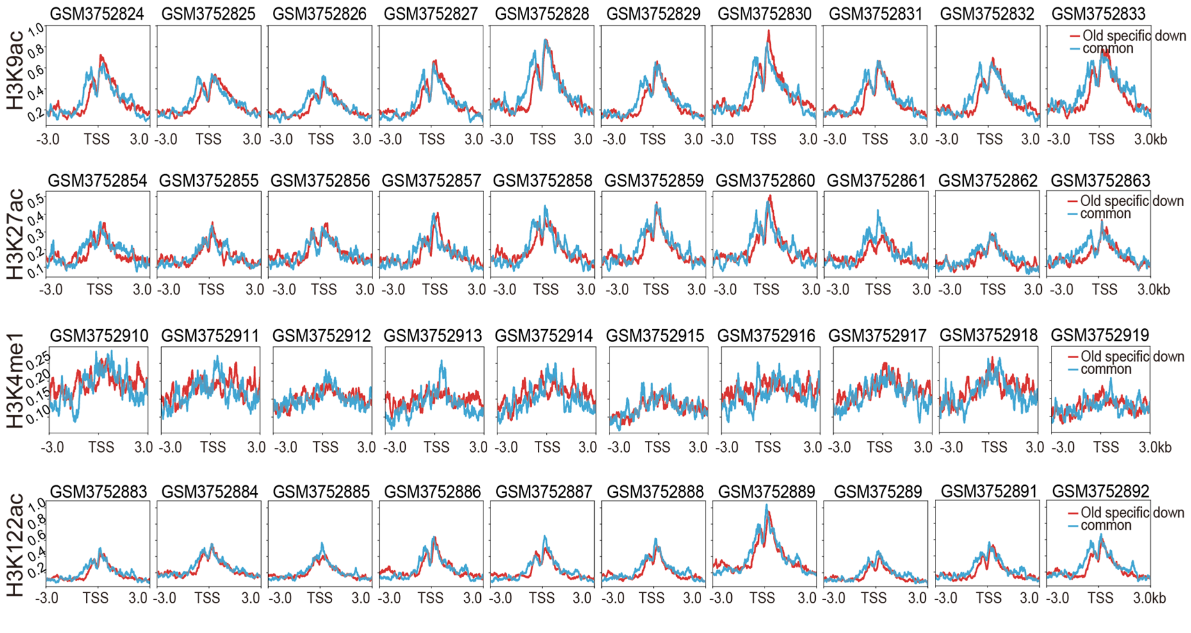

Figure S6. Four HMs signal distributions relative to ±3kb of the TSS between Old specific down-regulated genes and common down-regulated in the Old groups

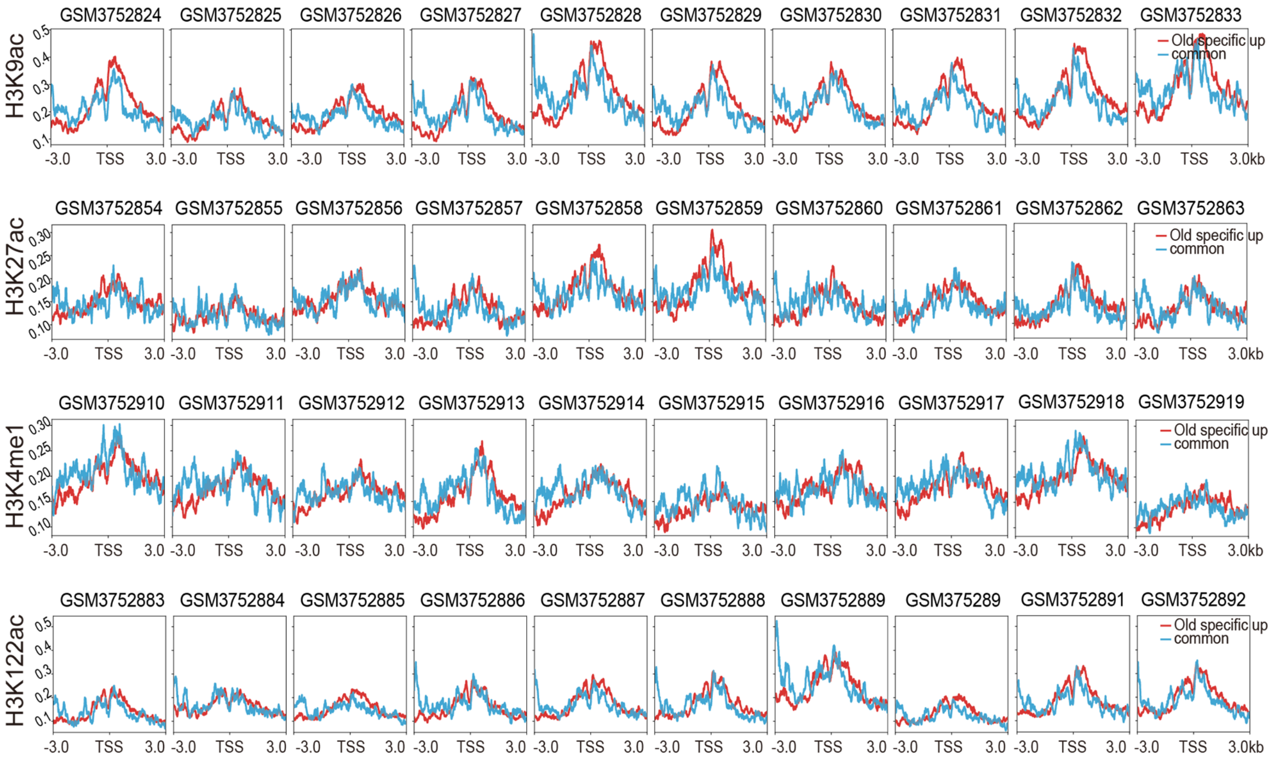

Figure S7. Four HMs signal distributions relative to ±3kb of the TSS between Old specific up-regulated genes and common up-regulated in the Old groups

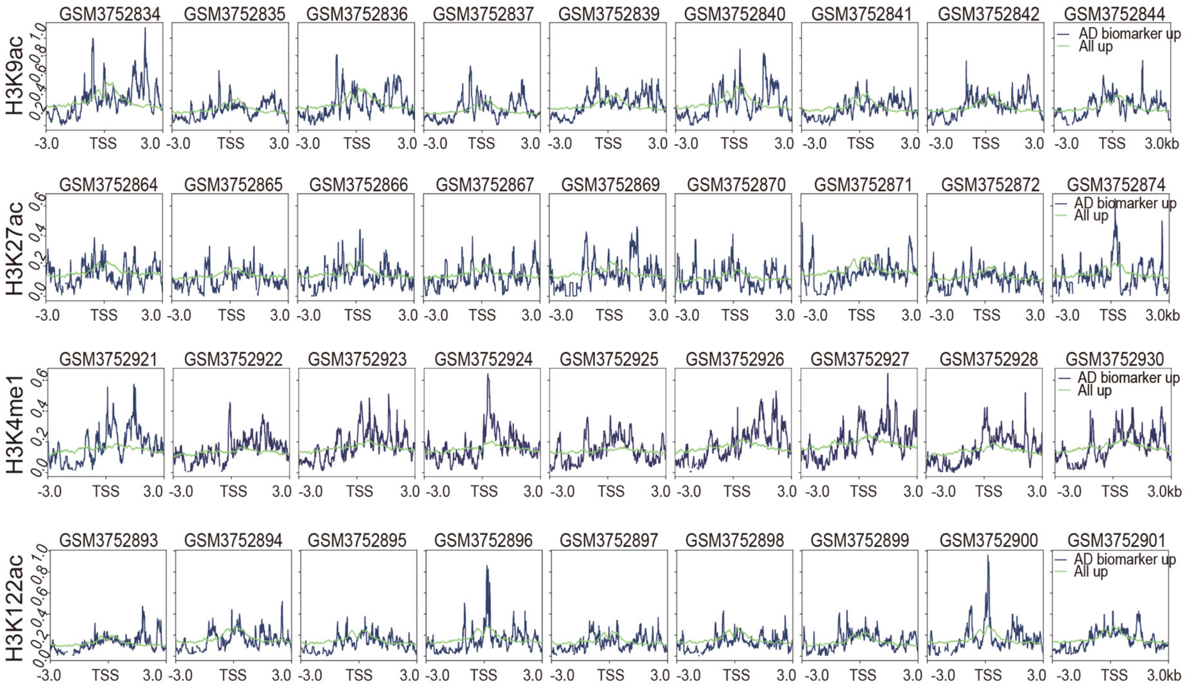

Figure S8. Four HMs signal distributions relative to ±3kb of the TSS between AD up-regulated biomarker genes and all up-regulated in the AD groups

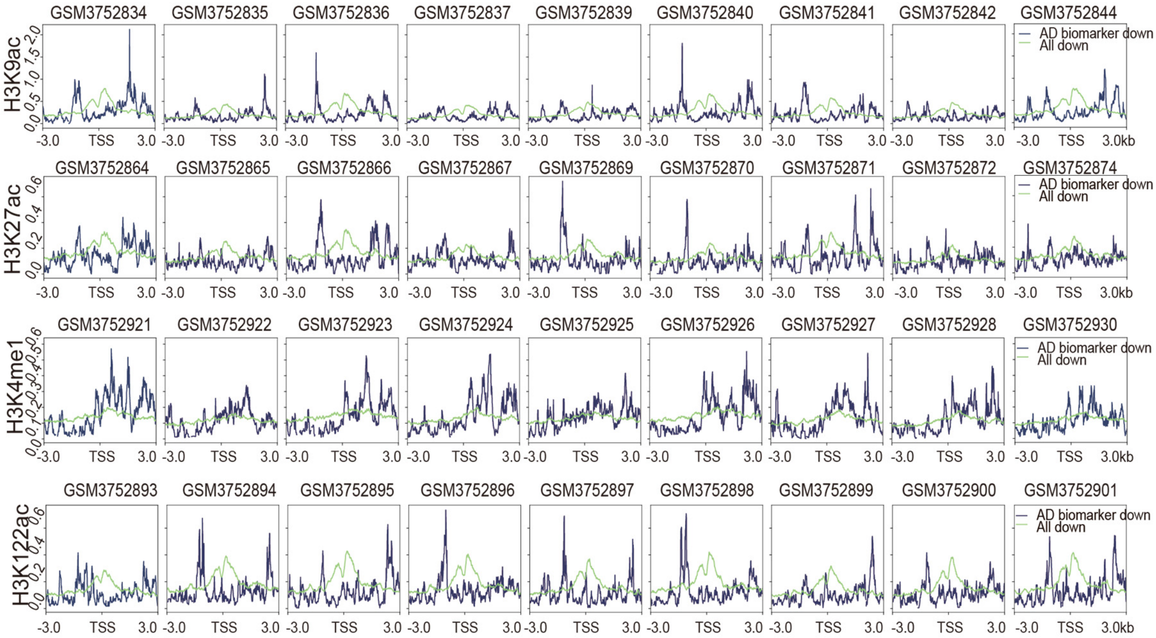

Figure S9. Four HMs signal distributions relative to ±3kb of the TSS between AD down-regulated biomarker genes and all down-regulated in the AD groups
